# CD4^+^ T Cell Subsets and *PTPN22* as Novel Biomarkers of Immune Dysregulation in Dilated Cardiomyopathy

**DOI:** 10.3390/ijms26167806

**Published:** 2025-08-13

**Authors:** Xinyu Zhang, Junteng Zhou, Yu Kang, Xiaojing Chen, Zixuan Yang, Yingjing Xie, Ting Liu, Xiaojing Liu, Qing Zhang

**Affiliations:** 1 Department of Cardiology, West China Hospital of Sichuan University, Chengdu 610041, China; hxzhangxinyu2014@163.com (X.Z.); kangyu_46@scu.edu.cn (Y.K.); chenxiaojing_058@163.com (X.C.); yzx350577974@163.com (Z.Y.); xieyingjing163@163.com (Y.X.); liutingmed@163.com (T.L.); 2 Department of Health Management Center, General Practice Medical Center, West China Hospital of Sichuan University, Chengdu 610041, China; zhoujunteng@scu.edu.cn; 3 Department of Cardiology and Laboratory of Cardiovascular Diseases, West China Hospital of Sichuan University, Chengdu 610041, China

**Keywords:** dilated cardiomyopathy, CD4^+^ T cells, *PTPN22*, immune dysregulation, gene expression profiling, weighted gene coexpression network analysis, machine learning

## Abstract

Recent multiomics advancements have improved our understanding of immune dysregulation in dilated cardiomyopathy (DCM). However, specific immune cell subsets and their regulatory genes are still ambiguous. This study aimed to explore immune cell imbalances and regulatory genes in DCM, discover diagnostic biomarkers, and identify potential therapeutic targets. Immune cell infiltration in DCM patients was quantified via deconvolution algorithms and single-cell RNA sequencing. Flow cytometry validation in 40 DCM patients and 40 healthy controls confirmed a notable increase in CD4^+^ effector memory T cells (CD4^+^ TEM cells) in DCM patients. Differential expression analysis of the GSE101585 dataset revealed 1783 genes. Weighted gene coexpression network analysis (WGCNA) identified a core immune-regulatory gene set, and protein–protein interaction (PPI) analysis highlighted 36 hub genes. Machine learning cross-validation identified four diagnostic biomarkers (*LRRTM4*, *PTPN22*, *FAM175B*, and *PROM2*) whose transcriptional changes had been validated by qPCR. Among these genes, *PTPN22* was strongly correlated with CD4^+^ TEM cell abundance. Additionally, DSigDB analysis predicted 87 potential therapeutic drugs, with *PTPN22* being the target of the most drugs. This study reveals a CD4^+^ T cell subset-centered immunoregulatory network in DCM, identifying novel diagnostic biomarkers and druggable targets to guide precision immunomodulatory strategies for DCM management.

## 1. Introduction

Dilated cardiomyopathy (DCM) is a cardiac disease characterized by cardiac chamber dilation and myocardial systolic dysfunction, leading to heart failure and the potential need for heart transplantation [1]. Its etiology, potentially linked to genetic factors, myocarditis, alcohol/toxin exposure, and systemic diseases [2], remains incompletely understood. Recent research suggests that immune system activation may play a pivotal role in connecting these pathogenic factors to myocardial damage.

Abnormalities in the immune system play a significant role in DCM through the myocardial inflammatory response and fibrosis mediated by immune cell activation [3]. Injury triggers the recruitment of proinflammatory infiltrates, such as CD8^+^ T cells and M1 macrophages, and promotes T cell activation [4]. Infiltrated macrophages and T cells trigger myocardial fibrosis by releasing IL-1β and TNF-α [5]. Key bottlenecks in DCM research include an incomplete understanding of dynamic changes in peripheral immune cell subsets and their regulatory gene associations with myocardial injury. Studies often focus on single immune components and lack an integrated analysis of the immune microenvironment, hindering a comprehensive understanding of the immunopathological mechanisms of DCM [5]. The development of targeted therapeutic strategies for the DCM immune microenvironment is significantly lagging behind established strategies in tumor immunotherapy [6], impeding precision treatment advancements.

This study utilized the peripheral immune characteristics of DCM patients and integrated multiomics data and machine learning algorithms to analyze immune cell infiltration patterns and regulatory networks. By identifying differentially expressed genes (DEGs), constructing coexpression networks, and identifying hub genes involved in protein–protein interactions (PPIs), along with machine learning cross-validation, this study aims to identify core genes with diagnostic and immune regulatory functions and investigate potential targeted drugs.

## 2. Results

### 2.1. Immune Alterations in Dilated Cardiomyopathy

#### 2.1.1. Immune Alterations via Deconvolution Algorithms

**Figure 1:**
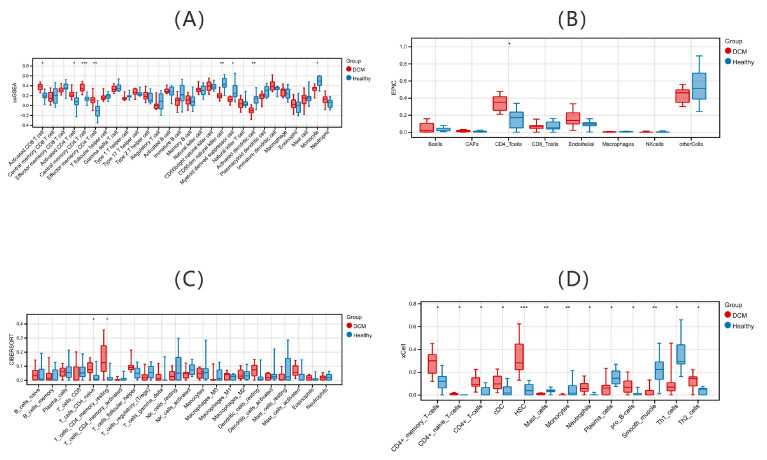
Peripheral blood immune landscape in dilated cardiomyopathy (DCM) patients versus healthy controls. Boxplot of immune cell composition evaluated by (**A**) ssGSEA, (**B**) EPIC, (**C**) CIBERSORT, and (**D**) xCell in the DCM and healthy groups. * *p* < 0.05, ** *p* < 0.01, *** *p* < 0.001. Abbreviations: HSCs: hematopoietic stem cells; CAFs: cancer-associated fibroblasts; NK cells: natural killer cells; cDCs: conventional dendritic cells; Tregs: regulatory T cells; Th1 cells: type 1 helper T cells; Th2 cells: type 2 helper T cells; pDCs: plasmacytoid dendritic cells.

This study utilized four deconvolution algorithms (ssGSEA, EPIC, xCell, and CIBERSORT) on transcriptome data from the bulk RNA-seq dataset GSE101585 training set to assess the immune microenvironment in DCM patients versus healthy controls (Figure 1). Key findings highlight the reprogramming of CD4^+^ T cell subsets: ssGSEA revealed significant enrichment of activated CD4^+^ T cells, central memory CD4^+^ T cells (CD4^+^ TCM), effector memory CD4^+^ T cells (CD4^+^ TEM), and activated CD8^+^ T cells in the DCM group (*p* < 0.05). In contrast, the numbers of CD56dim NK cells, myeloid-derived suppressor cells, activated dendritic cells, and monocytes were notably lower than those in the control group. Cross-algorithm validation further strengthened the central position of CD4^+^ T cells: EPIC and CIBERSORT analyses revealed significant upregulation of CD4^+^ T cells, naive CD4^+^ T cells, and memory resting CD4^+^ T cells in DCM patients. xCell analysis specifically revealed the accumulation of memory CD4^+^ T cells, naive CD4^+^ T cells, conventional dendritic cells (cDCs), and hematopoietic stem cells (HSCs) in the DCM group.

#### 2.1.2. Immune Alterations via Single-Cell RNA Sequencing

**Figure 2:**
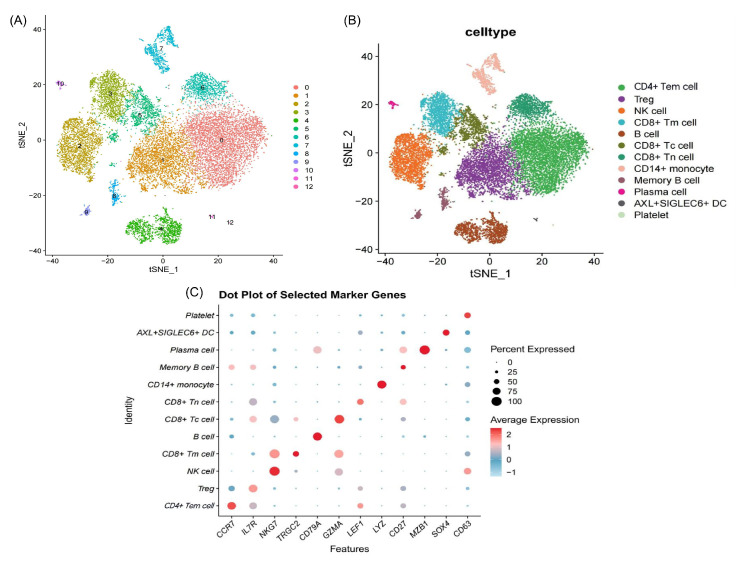
t-SNE clustering and marker gene expression of immune cell subtypes. (**A**) t-SNE plot of cell clusters based on unsupervised clustering. (**B**) t-SNE plot of annotated immune cell types. (**C**) Expression of specific genes across different cell types. The size of each dot indicates the percentage of gene expression in the corresponding cell, and the color intensity reflects the average expression level. Abbreviations: CD4^+^ Tem cells: CD4^+^ effector memory T cells; CD8^+^ Tc cells: CD8^+^ cytotoxic T cells; CD8^+^ Tn cells: naive CD8^+^ T cells; CD8^+^ Tm cells: CD8^+^ memory T cells; DCs: dendritic cells.

We obtained single-cell RNA sequencing (scRNA-seq) data GSE145154 for patients with DCM and healthy controls from the public GEO database. Following quality control, normalization, and batch correction, the processed dataset demonstrated high quality, as evidenced by clear separation of distinct cell populations (Supplemental Figure S1). t-SNE clustering analysis resolved all the cells into 13 distinct clusters (Figure 2A). Annotation on the basis of marker gene expression revealed 12 distinct cell types within these clusters (Figure 2B), with the marker genes for each cell type listed in Figure 2C and Supplemental Figure S2.

**Figure 3:**
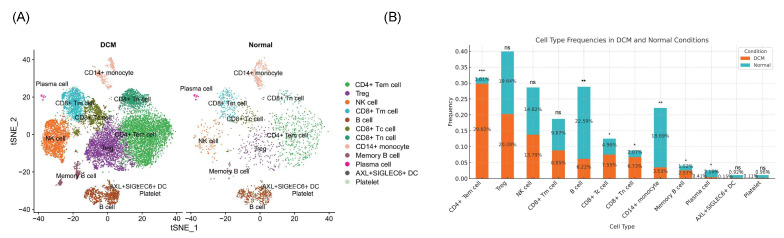
Immune cell landscape and frequency comparison between DCM patients and controls. (**A**) t-SNE plot of immune cell clusters in DCM patients and controls. (**B**) Bar plot of cell type frequencies in DCM patients and controls. * *p* < 0.05, ** *p* < 0.01, *** *p* < 0.001.

We analyzed cell type expression levels across sample groups to determine immune cell distribution in the peripheral blood of DCM patients versus healthy controls. The results (Figure 3) revealed that B cells, CD14^+^ monocytes, and plasma cells significantly decreased in DCM patients (*p* < 0.05), whereas CD4^+^ TEMs, CD8^+^ cytotoxic T cells (CD8^+^ Tc cells), CD8^+^ naive T cells (CD8^+^ Tn cells), and memory B cells significantly increased (*p* < 0.05). Remarkably, the percentage of CD4^+^ TEMs in DCM patients reached 29.82%, whereas it reached 1.81% in controls (*p* < 0.001), highlighting their crucial role in the DCM immune response and pathology.

**Figure 4:**
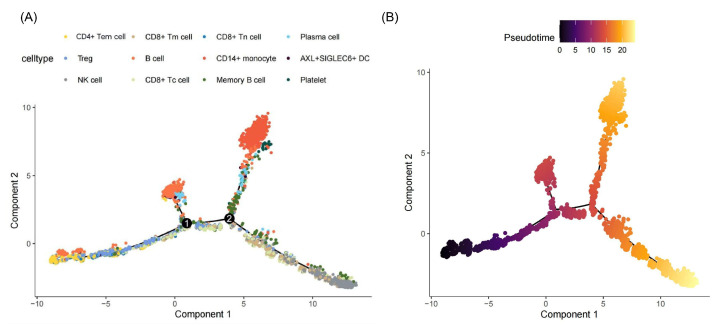
Trajectory analysis of immune cell differentiation and pseudotime ordering. (**A**) t-SNE plot of immune cell clusters and trajectories in the component space. (**B**) Pseudotime analysis of immune cell trajectories. The black line indicates the inferred developmental path, with numbers “1” and “2” representing key branch points where cells diverge into distinct lineages.

Pseudotime analysis indicated that CD4^+^ TEM cells predominantly occupied the early stages of the trajectory, which was situated on the left. As pseudotime advanced, the number of CD4^+^ TEMs decreased, suggesting that these cells were converted into other effector cells during differentiation (Figure 4).

### 2.2. Flow Cytometry Validates Algorithm-Predicted T Cell Shifts

An independent clinical cohort (40 DCM patients vs. 40 healthy controls) was subjected to flow cytometry analysis to validate immune cell level estimation via a deconvolution algorithm. The DCM patients (average age: 46.81 ± 13.1 years) and healthy controls (average age: 49.36 ± 8.2 years) were not significantly different in terms of age (*p* = 0.278). The sex distribution was similar between the groups (*p* = 0.352). The average disease duration in DCM patients was 0.15 ± 0.07 years, with 97.7% being newly diagnosed. All patients were NYHA class I–II. Etiologically, 38 patients had idiopathic DCM, and 2 had alcohol-related DCM. The comorbidities included hypertension (three patients), atrial fibrillation (three patients), and complete left bundle branch block (six patients).

**Figure 5:**
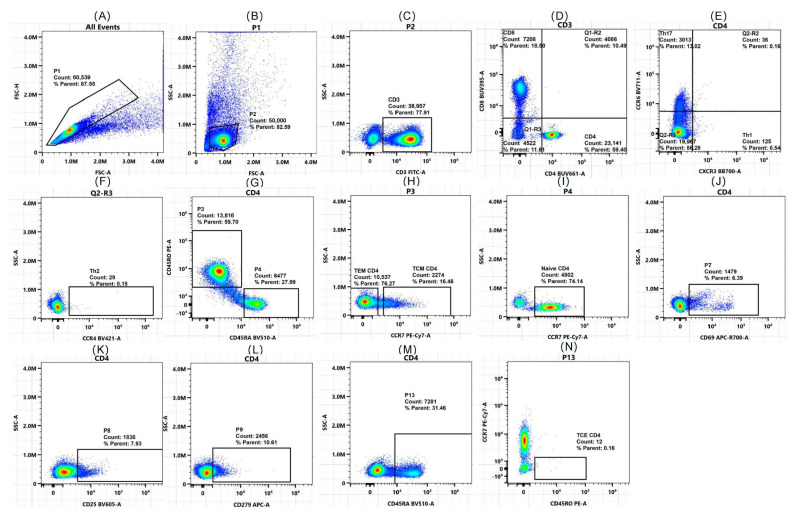
Flow cytometry analysis of CD4^+^ T cell subsets in the peripheral blood of patients with DCM (*n* = 40). (**A**) All events: initial gating on forward scatter (FSC-A) and side scatter (SSC-A) to exclude debris. (**B**) Lymphocyte gate (P1): selection of the lymphocyte population via FSC-A vs. SSC-A. (**C**) Single gate (P2): exclusion of doublets by gating on FSC-H vs. FSC-A. (**D**) T cell gate (P3): identification of CD3^+^ T cells and further separation into CD4^+^ and CD8^+^ subsets. (**E**) Th1/Th17 subsets: identification of Th1 and Th17 cells within the CD4^+^ T cell population on the basis of CXCR3 and CCR6 expression. (**F**) Q2-R3: identification of Th2 cells on the basis of CCR4 expression. (**G**) CD4^+^ naive and memory T cells: Differentiation of CD4^+^ naive and memory T cells on the basis of CD45RA and CCR7 expression. (**H**) CD4^+^ TCM and TEM cells: further characterization of memory CD4^+^ T cells via CCR7 and CD45RA. (**I**) CD4^+^ naive T cells: gating of CD4^+^ naive T cells (CD45RA+CCR7+). (**J**) CD69+CD4^+^ T cells: expression of the early-activation marker CD69. (**K**) CD25+CD4^+^ T cells: expression of the late-activation marker CD25. (**L**) PD-1 (CD279)+CD4^+^ T cells: expression of the exhaustive marker PD-1. (**M**) Additional CD4^+^ subsets: gating for additional CD4^+^ T cell subsets on the basis of CD45RA. (**N**) CD4^+^ TCE cells: differentiation of terminal effector CD4^+^ T cells on the basis of CCR7 and CD45RO expression. The colors represent different cell types.

**Figure 6:**
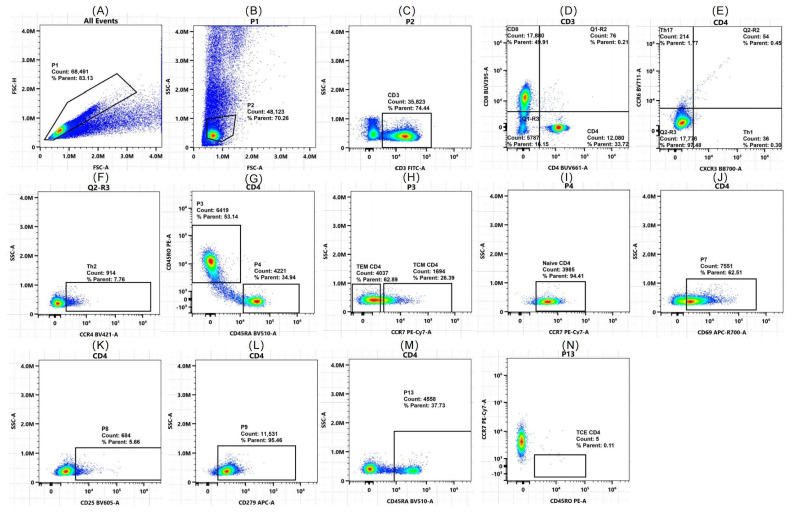
Flow cytometry analysis of CD4^+^ T cell subsets in peripheral blood from healthy controls (*n* = 40). (**A**) All events: initial gating on forward scatter (FSC-A) and side scatter (SSC-A) to exclude debris. (**B**) Lymphocyte gate (P1): selection of the lymphocyte population via FSC-A vs. SSC-A. (**C**) Single gate (P2): exclusion of doublets by gating on FSC-H vs. FSC-A. (**D**) T cell gate (P3): identification of CD3^+^ T cells and further separation into CD4^+^ and CD8^+^ subsets. (**E**) Th1/Th17 subsets: identification of Th1 and Th17 cells within the CD4^+^ T cell population on the basis of CXCR3 and CCR6 expression. (**F**) Q2-R3: identification of Th2 cells on the basis of CCR4 expression. (**G**) CD4^+^ naive and memory T cells: Differentiation of CD4^+^ naive and memory T cells on the basis of CD45RA and CCR7 expression. (**H**) CD4^+^ TCM and TEM cells: further characterization of memory CD4^+^ T cells via CCR7 and CD45RA. (**I**) CD4^+^ naive T cells: gating of CD4^+^ naive T cells (CD45RA+CCR7+). (**J**) CD69+CD4^+^ T cells: expression of the early-activation marker CD69. (**K**) CD25+CD4^+^ T cells: expression of the late-activation marker CD25. (**L**) PD-1 (CD279)+CD4^+^ T cells: expression of the exhaustive marker PD-1. (**M**) Additional CD4^+^ subsets: gating for additional CD4^+^ T cell subsets on the basis of CD45RA. (**N**) CD4^+^ TCE cells: differentiation of terminal effector CD4^+^ T cells on the basis of CCR7 and CD45RO expression. The colors represent different cell types.

For the 12-color flow cytometry analysis (Table 1), peripheral blood T lymphocytes and subsets were compared between the case group (Figure 5) and the control group (Figure 6). The proportion of CD4^+^ T cells among CD3^+^ T cells was notably greater in DCM patients than in healthy controls (median [IQR]: 54.15% [41.87–61.47] vs. 49.55% [4.66–56.4], *p* = 0.043). Subgroup analysis revealed a greater proportion of CD4^+^ TEM cells than in the control group (IQR: 7.54% [4.57–18.85] vs. 3.96% [1.53–5.30], *p* = 0.001), which aligns with predictions from the ssGSEA algorithm. Additionally, CD4^+^ TCM cells (21.63 ± 10.93% vs. 14.19 ± 7.81%, *p* = 0.013) and early-activated CD4^+^ T cells (3.59% vs. 1.2%, *p* = 0.001) increased significantly, whereas naive CD4^+^ T cells decreased (17.5% vs. 27.8%, *p* = 0.039), supporting a shift toward an activated/memory CD4^+^ T cell phenotype.

For CD8^+^ T cells, the DCM group presented a significant increase in the proportion of CD8^+^ T cells (38.74 ± 15.19% vs. 24.48 ± 8.34%, *p* < 0.001), which was consistent with the prediction by the ssGSEA algorithm. The proportion of early-activated CD8^+^ T cells also increased in the DCM group (11.65% vs. 4.61%, *p* = 0.012), but there were no significant differences in CD8^+^ TCE cells or CD8^+^ TEM cells. Notably, there were no significant differences in exhausted (PD-1+) or late-activated CD4^+^ or CD8^+^ T cells between the two groups in the clinical cohort (*p* > 0.05), indicating that the T cell abnormalities in DCM patients may be driven predominantly by early activation rather than terminal exhaustion.

### 2.3. Identification and Functions of Differentially Expressed Genes

**Figure 7:**
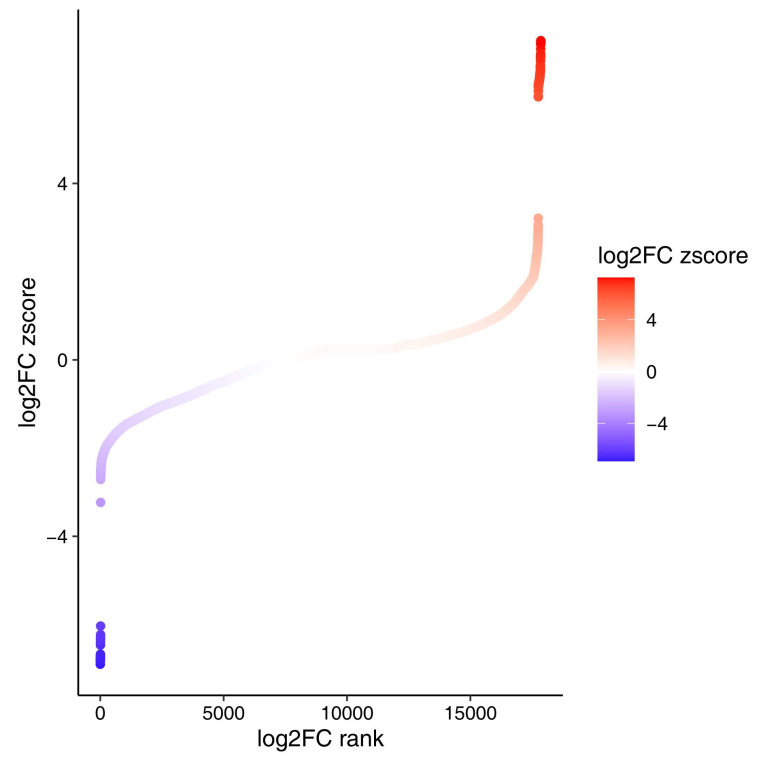
Differentially expressed gene ranking plot. X-axis: log_2_FC rank; Y-axis: log_2_FC z score, representing the standardized log_2_FC values. log_2_FC z score = (log_2_FC − µ)/σ (where µ represents the mean log_2_FC and σ the standard deviation across all genes). Red: upregulated genes (positive log_2_FC z scores); blue: downregulated genes (negative log_2_FC z scores).

Differential expression analysis of the GSE101585 dataset revealed 1783 DEGs between the disease and control groups according to strict criteria (*p* < 0.05 and |log_2_FC| > 2). Among these genes, 348 genes were upregulated and 1435 genes were downregulated in the DCM group. Literature mining and DisGeNET annotation revealed that 621 DEGs (34.8%) were associated with DCM mechanisms. In Figure 7, the gene expression ranking plot reveals a cluster of highly upregulated genes with log_2_FC z scores above 5, while a distinct group of strongly downregulated genes is found below −6. These patterns highlight profound transcriptomic shifts in the blood of DCM patients, with a subset of genes dramatically activated or suppressed compared with those in controls.

### 2.4. Analysis of Immune-Related Gene Networks

**Figure 8:**
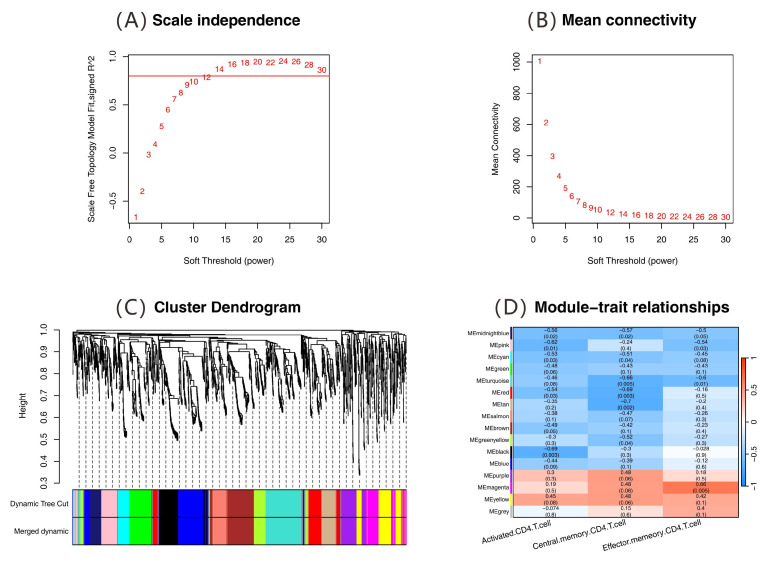
Identification of immune-related coexpression modules via weighted gene coexpression network analysis (WGCNA). (**A**) Scale-free topology fit analysis: relationship between soft-thresholding power and scale-free topology fit index (R^2^). The red lines represent the selected threshold for network construction. (**B**) Mean connectivity analysis: negative correlation between soft-thresholding power and network connectivity. (**C**) Hierarchical clustering dendrogram of coexpression modules. The gray symbol indicates genes not assigned to modules. The color bands indicate dynamic tree cut results. (**D**) Module–trait correlation heatmap: correlations between coexpression modules and CD4^+^ T cell subsets. The colors represent the strength of the correlation, with blue indicating negative and red indicating positive correlations.

Weighted gene coexpression network analysis (WGCNA) identified 16 coexpression modules from 1783 DEGs. Through the validation of the scale-free network, the soft threshold parameter was determined to be 14 (soft power = 14). As shown in Figure 8A, the scale-free topological criterion was met when R^2^ > 0.8. Moreover, Figure 8B shows that the mean value of the adjacency function tended to be stable at this threshold. A hierarchical clustering tree was built with a deep split value (deepSplit = 2), minimum genes in a module (minModuleSize = 50), and a module merging similarity threshold (mergeCutHeight = 0.25, 75% correlation). Sixteen coexpression modules were identified (Figure 8C). Correlation analysis linked module eigengenes with the ssGSEA scores of three CD4^+^ T cell subsets. The magenta module was notably correlated with CD4^+^ TEM cells (r = 0.66, *p* = 0.005; Figure 8D). This module, containing 104 genes, was designated the core gene set for immune regulation for further functional analysis.

**Figure 9:**
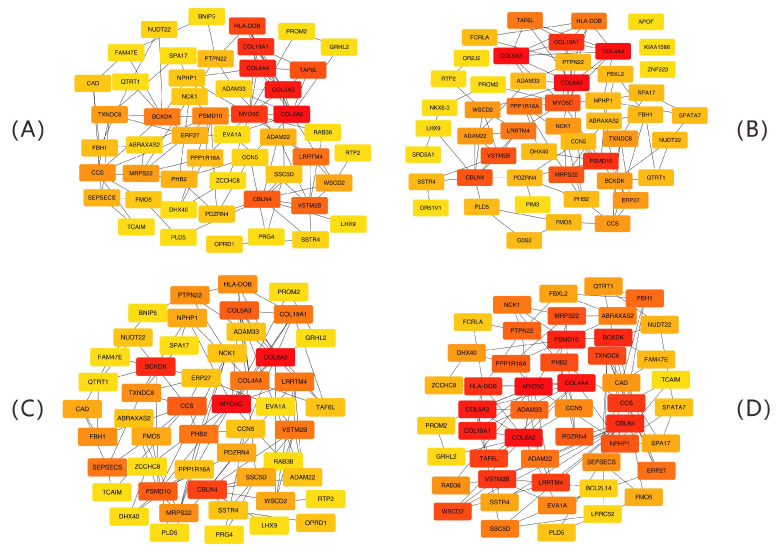
Key hub gene identification via topological algorithms. (**A**) Maximal clique centrality (MCC). (**B**) Maximum neighborhood component (MNC). (**C**) Degree centrality. (**D**) Edge percolated component (EPC). Node color gradient: hub gene significance (red = highest, yellow = lowest). Connecting lines: protein–protein interactions (PPIs) with confidence scores ≥ 0.15.

**Figure 10:**
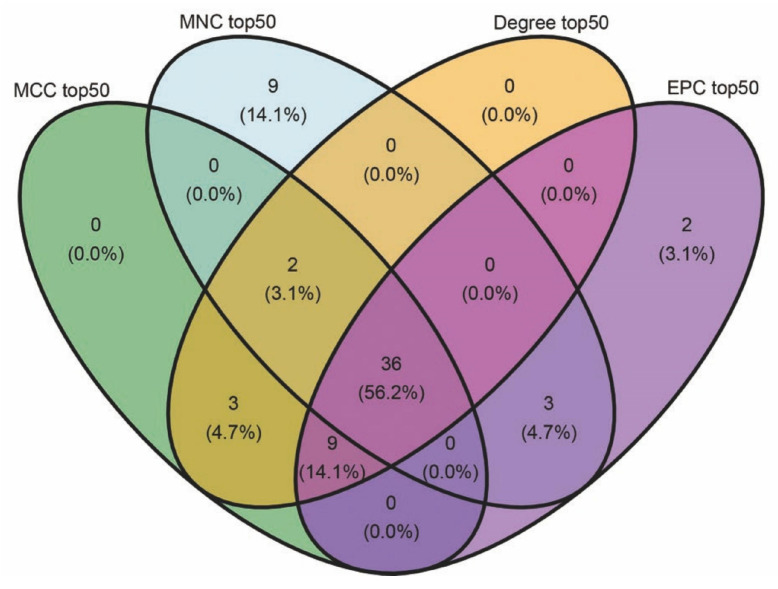
Key hub gene overlap via topological algorithms. Venn diagram showing the intersection of genes identified by the four algorithms (MCC, MNC, degree, and EPC). The numbers indicate unique and overlapping genes; percentages reflect the proportion relative to the total number of hub genes identified per algorithm.

A total of 104 immune regulatory module genes were analyzed via the STRING database to create a PPI network with a minimum confidence of 0.15. Isolated nodes were excluded. Figure 9 displays interaction networks of the top 50 hub genes identified by four topological algorithms (MCC, MNC, degree, and EPC). A node color shift from yellow to red signifies gene importance, with key genes selected on the basis of a node degree of ≥20. A Venn diagram (Figure 10) analysis revealed that 36 core hub genes were consistently identified by all four algorithms, representing 56.2% of the total genes. Among the four algorithms, both *COL19A1* and *HLA-DOB* exhibited high connectivity, indicating significant network centrality characteristics. Specifically, *COL19A1* presented high connectivity in both the MCC (degree = 32, ranked fourth) and EPC (degree = 30, ranked fifth) algorithms. It formed a high-density interaction subnetwork (18 nodes, average node degree = 23.5 ± 2.8, clustering coefficient = 0.68 ± 0.05) with *HLA-DOB* in the STRING PPI network (confidence > 0.7). Functional enrichment analysis revealed that this interaction network was significantly enriched in antigen presentation (GO:0048002, FDR = 1.3 × 10^−7^) and the T cell receptor signaling pathway (KEGG:04660, FDR = 4.1 × 10^−5^).

### 2.5. Results of Feature Gene Screening

**Figure 11:**
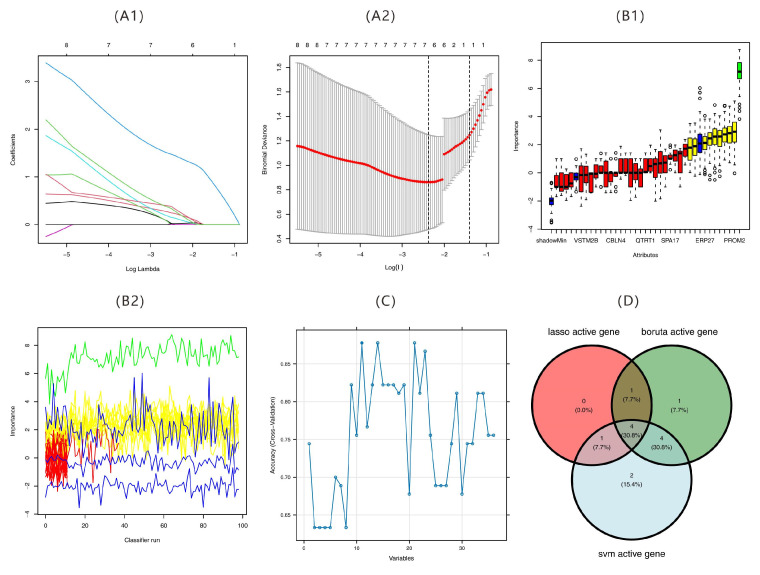
Machine learning-based identification of hub genes. (**A**) LASSO regression model analysis: (**A1**) coefficient profiles of features along the regularization path in the least absolute shrinkage and selection operator (LASSO) regression model; (**A2**) cross-validation error curve for LASSO model selection. (**B**) Boruta feature selection: (**B1**) feature importance ranking from the Boruta algorithm. Different box colors represent the evaluation of variable importance: green for confirmed features, yellow for tentative features, and red/blue for rejected or shadow features. Black dots indicate outliers; (**B2**) classification performance across multiple runs. (**C**) Support vector machine recursive feature elimination (SVM-RFE) feature selection: cross-validation accuracy curve for variable selection via the SVM-RFE algorithm. (**D**) Overlap of active genes: Venn diagram illustrating the overlap of active genes identified by the LASSO, Boruta, and SVM algorithms.

On the basis of these 36 core hub genes identified through the PPI network, machine learning algorithms were employed to further select the genes most predictive of the target phenotype. This study utilized a multi-algorithm fusion strategy to identify core gene combinations associated with a target phenotype (Figure 11). Initially, the LASSO regression algorithm was employed to select six genes (*LRRTM4*, *PTPN22*, *FBXO18*, *WISP2*, *FAM175B*, and *PROM2*) with high predictive value (Figure 11A1,A2). The Boruta algorithm with a random forest model subsequently identified ten significant genes (*COL19A1*, *LRRTM4*, *WSCD2*, *ERP27*, *ADAM22*, *PTPN22*, *ADAM33*, *WISP2*, *FAM175B*, and *PROM2*), with *PROM2* being the most important (Figure 11B1,B2). The SVM-RFE algorithm was then used to retain 11 key genes by eliminating low-weight features (Figure 11C). Through intersection analysis of the three gene sets via a Venn diagram, *LRRTM4*, *PTPN22*, *FAM175B*, and *PROM2* were determined as the four core candidate genes verified across algorithms (Figure 11D).

### 2.6. Validation of Feature Genes

#### 2.6.1. Validation of Feature Genes by qPCR

**Figure 12:**
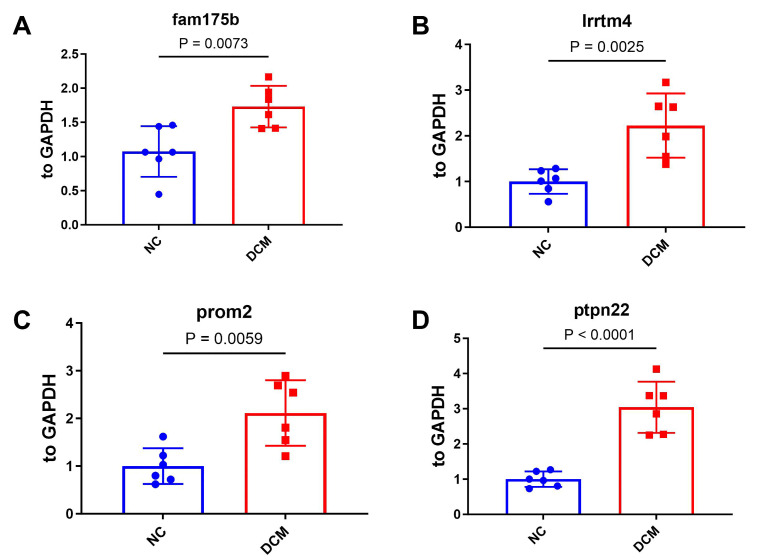
Gene expression analysis of key markers in CD4^+^ TEMs from human peripheral blood. Quantitative PCR (qPCR) analysis of (**A**) *FAM175B*, (**B**) *LRRTM4*, (**C**) *PROM2*, and (**D**) *PTPN22* mRNA expression (normalized to GAPDH) in peripheral blood CD4^+^ TEM cells from six DCM patients and six healthy controls.

Quantitative PCR (qPCR) validation in peripheral blood CD4^+^ TEM cells from six DCM patients and six healthy controls revealed significant upregulation of four signature genes (Figure 12). *FAM175B* exhibited a 1.61-fold increase (*p* = 0.0073), *LRRTM4* presented a 2.23-fold increase (*p* = 0.0025), *PROM2* presented a 2.11-fold increase (*p* = 0.0059), and *PTPN22* presented the most pronounced increase, 3.04-fold (*p* < 0.0001), confirming robust transcriptional alterations in DCM.

#### 2.6.2. Validation of the Diagnostic Efficacy and Immune Correlation of the Feature Genes

**Figure 13:**
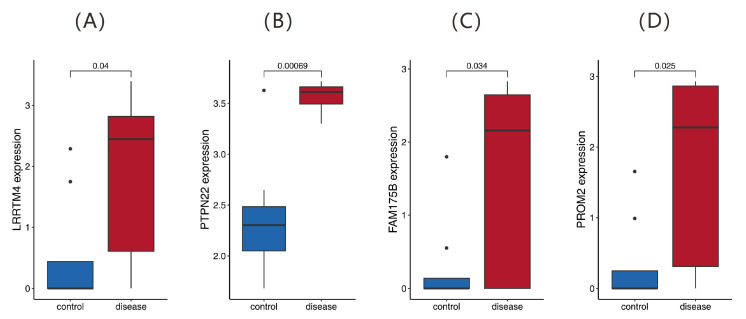
Expression profiling of candidate biomarker genes. (**A**–**D**) Box plots showing the differential expression of four genes (*LRRTM4*, *PTPN22*, *FAM175B*, and *PROM2*) between the disease and control groups, with *p* values indicated above. The black dots represent outliers beyond 1.5 times the interquartile range.

**Figure 14:**
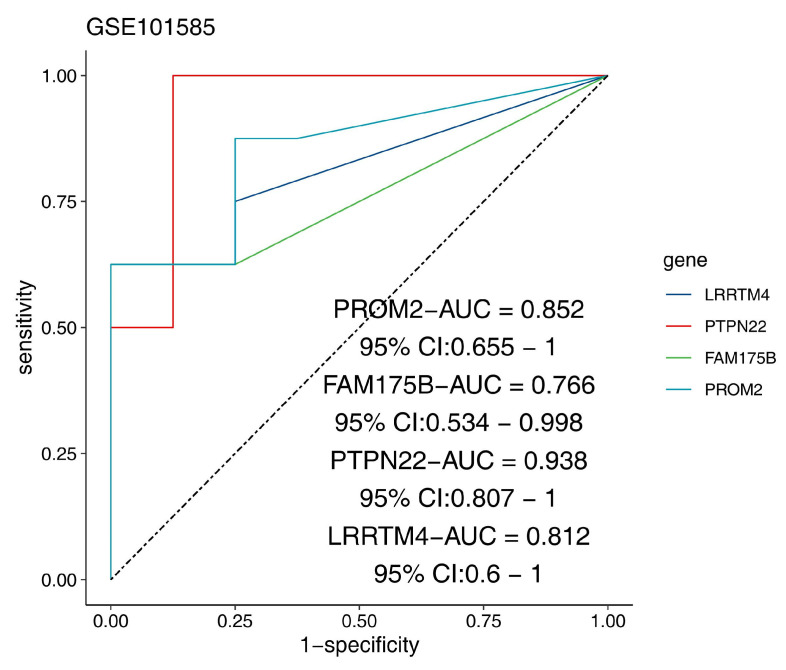
Diagnostic potential of candidate biomarker genes. Receiver operating characteristic (ROC) curve analysis of the GSE101585 dataset demonstrated the high diagnostic accuracy of all four genes (*LRRTM4*, *PTPN22*, *FAM175B*, and *PROM2*). Abbreviations: AUC: area under the curve; CI: confidence interval.

*LRRTM4* (AUC = 0.812), *PTPN22* (AUC = 0.938), *FAM175B* (AUC = 0.766), and *PROM2* (AUC = 0.852) were identified as core diagnostic markers for distinguishing DCM patients from healthy controls because of their stable differential expression (|log2FC| > 1, FDR < 0.05, Cohen’s d > 0.8) and excellent disease classification performance (Figure 13). These genes were identified as core diagnostic markers for distinguishing DCM patients from healthy controls. Among them, *PTPN22* showed particularly outstanding diagnostic efficacy, with an AUC as high as 0.938 and a Youden index of 0.87 (Figure 14).

**Figure 15:**
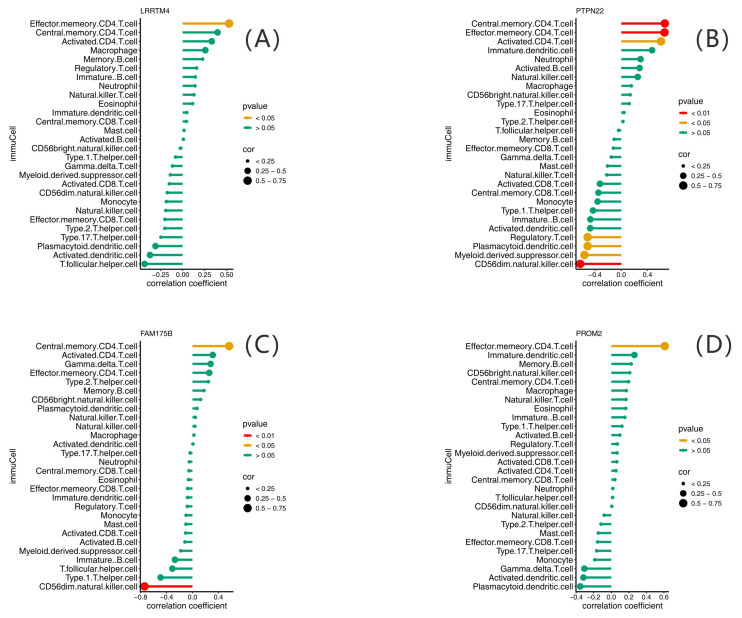
Association of diagnostic genes with immune cell infiltration. Lollipop plots illustrating correlations between immune cells and (**A**) *LRRTM4*, (**B**) *PTPN22*, (**C**) *FAM175B*, and (**D**) *PROM2*, respectively. The point size represents the correlation strength, and the color indicates the significance level.

**Figure 16:**
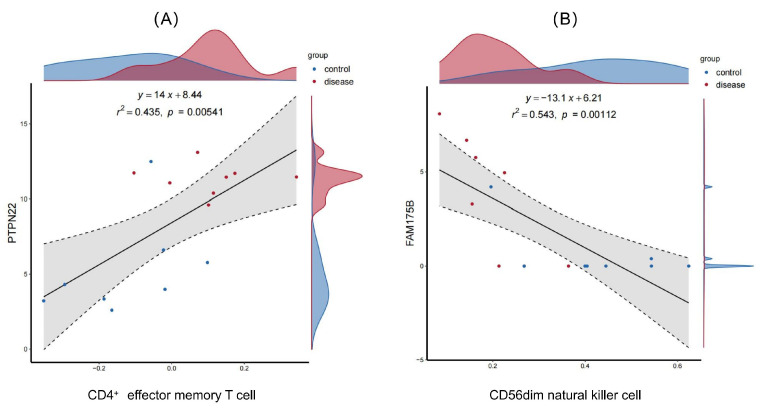
Immune correlates of diagnostic genes. (**A**) Scatter plot demonstrating a positive correlation between *PTPN22* and effector memory CD4^+^ T cells (r^2^ = 0.435, *p* = 0.005). (**B**) Scatter plot showing a negative correlation between *FAM175B* and CD56dim NK cells (r^2^ = 0.543, *p* = 0.001).

Pearson correlation was used to assess the correlations of diagnostic genes with immune cell infiltration. The Lollipop plots in Figure 15 display the correlations of *LRRTM4*, *PTPN22*, *FAM175B*, and *PROM2* with immune cells. Eleven gene pairs correlated significantly with immune cell infiltration scores (*p* < 0.05). Notably, *PTPN22* exhibited a strong positive correlation with CD4^+^ TEM cells (r = 0.66, r^2^ = 0.435, *p* = 0.005), indicating a role in T cell memory differentiation. Conversely, *FAM175B* was significantly negatively correlated with CD56dim natural killer cells (r = −0.74, r^2^ = 0.543, *p* = 0.001) (Figure 16), suggesting a potential impact on disease progression via the suppression of NK cell function.

### 2.7. Drug Target Prediction

**Figure 17:**
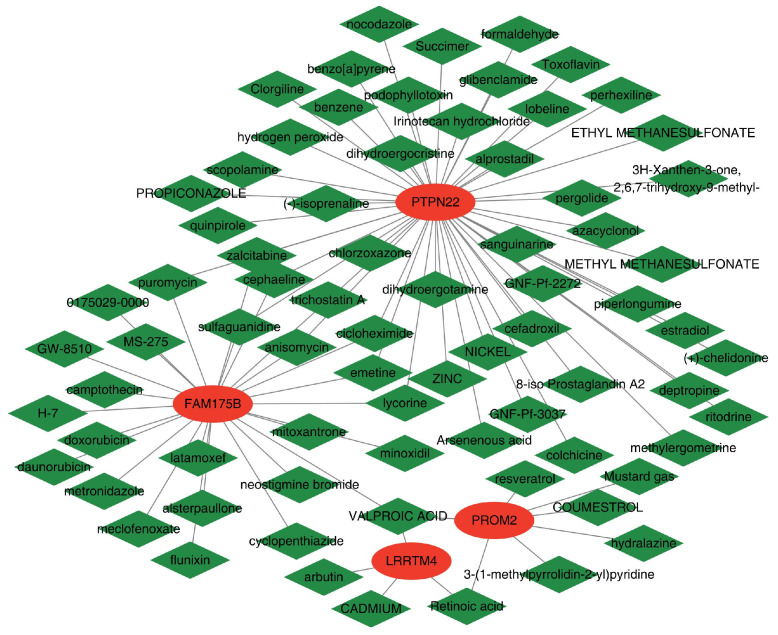
Drug–gene interaction network analysis leveraging the Drug Signatures (DSigDB) database. The network displays interactions between four diagnostic genes (red ellipses) and their potential drug compounds (green diamonds). The gray lines indicate the predicted drug–gene interactions.

Analysis of small-molecule drugs via the DSigDB database revealed distinct interaction patterns (Figure 17) with four diagnostic genes (*PTPN22*, *FAM175B*, *PROM2*, and *LRRTM4*). *PTPN22* has broad drug-binding potential and interacts with 50 small molecules, including nocodazole and glibenclamide. *FAM175B* interacts with 26 small molecules, mainly antitumor drugs such as doxorubicin and mitomycin. *PROM2* is associated with seven compounds, including colchicine. *LRRTM4* has specific interactions with four small molecules, including valproic acid and retinoic acid. *PTPN22* has the most significant multitarget intervention potential, while *FAM175B* and *PROM2* are linked to tumor and inflammation pathways, and the specific interactions of *LRRTM4* may guide precision treatment strategies. These distinct features support targeted treatment approaches for different genes.

## 3. Discussion

DCM diagnosis lacks early, noninvasive, specific markers and relies on clinical symptoms, imaging, and biopsy. Treatment focuses on improving heart failure symptoms without precise etiology-targeted strategies [7]. Previous studies have primarily explored diagnostic markers and therapeutic targets through gene expression analysis, overlooking the significant role of the immune microenvironment in DCM pathogenesis [8,9]. This study introduces a novel approach in which multiple algorithms are combined to analyze the immune microenvironment in DCM.

### 3.1. T Cell Activation and Immune Dysregulation in the Pathogenesis of DCM

This study employed bioinformatics analysis through single-cell sequencing and deconvolution algorithms, followed by flow cytometry validation, revealing significant enrichment of CD4^+^ T cell subsets in the peripheral blood of patients with DCM, which aligns closely with previous findings [10]. CD4^+^ T cells have been shown to activate cardiac fibroblasts by secreting proinflammatory cytokines, such as IL-17 and IFN-γ, which drive fibrosis and ventricular remodeling, thus playing crucial roles in the progression of DCM [11,12]. The xCell algorithm detected an increase in HSCs in the DCM group, indicating the possible involvement of abnormal bone marrow HSC mobilization in DCM development. Studies suggest that the inflammatory microenvironment can induce HSC differentiation into myeloid progenitor cells through the TNF-α/NF-κB pathway, worsening cardiac inflammation mediated by monocytes/macrophages [13]. The decrease in monocyte and dendritic cell levels in this study (results of ssGSEA) appears to conflict with the increase in HSCs, possibly owing to algorithm differences in cell type resolution. Metabolic changes during hematopoietic cell differentiation might underestimate early HSC levels via phenotype-based algorithms such as ssGSEA, while marker-sensitive algorithms such as xCell could better detect HSC accumulation [14].

The most striking finding from scRNA-seq was the dramatic expansion of CD4^+^ TEMs in DCM patients, which increased from 1.81% in healthy controls to 29.82% (*p* < 0.001). This nearly 16-fold increase highlights the pivotal role of CD4^+^ TEMs in DCM immunopathology, highlighting their crucial role in DCM inflammation and disease progression through sustaining myocardial inflammation, tissue fibrosis, cardiac damage, and dysfunction. Pseudotime trajectory analysis further suggests that CD4^+^ TEMs occupy early differentiation stages, with their subsequent decline coinciding with progression toward terminal effector states. This finding supports a model in which CD4^+^ TEMs serve as a precursor pool, differentiating into proinflammatory effector subsets that perpetuate myocardial inflammation [15]. Concomitant increases in CD8^+^ Tc cells and CD8^+^ Tn cells indicate broad T cell activation and recruitment. CD8^+^ Tc cells likely contribute directly to cardiomyocyte damage, whereas elevated Tn levels may reflect ongoing immune priming against cardiac antigens. Notably, the decrease in B cells, CD14^+^ monocytes, and plasma cells (*p* < 0.05) contrasts with the increase in memory B cells, suggesting a shift from innate and humoral immunity toward adaptive, cell-mediated responses [16].

This study validated the imbalance of T cell subsets in DCM patients through 12-color flow cytometry. CD4^+^ and CD8^+^ T cells exhibited shifts toward an activated/memory phenotype. CD4^+^ T cells shifted toward the TEM and TCM phenotypes, with a decrease in naive CD4^+^ T cells and an increase in early-activated (CD69^+^) CD4^+^ T cell subsets. No significant alterations were noted in the terminally exhausted (PD-1+) or late-activated (CD25^+^) subsets. The T cell immune disorder in DCM is likely due to early immune activation (CD69^+^ T cell expansion) rather than end-stage exhaustion (no significant increase in PD-1+/CD25^+^ T cells). This finding differs from Efthimiadis et al.’s previous findings [17]. The discrepancy in disease progression among the study groups may be attributed to differences in disease stages. The current cohort consists mainly of newly diagnosed DCM patients (with an average disease duration of 2 years) in a persistent immune response state. The reduced number of naive CD4^+^ T cells and increased number of memory T cells (TCM/TEM) suggest antigen-specific T cell clonal expansion in the early DCM stages rather than terminal exhaustion from chronic antigen exposure. CD8^+^ T cells display heightened early activation and expansion, yet terminal differentiation subsets and the exhaustion marker PD-1+ remain unaltered, suggesting that abnormalities are confined to early activation without advancing to terminal effector or exhaustion phases. This finding corresponds with recent findings of temporary CD8^+^ T cell activation without enduring myocardial infiltration during acute DCM [18]. CD8^+^ T cells without terminal exhaustion may harm cardiomyocytes through cytotoxic granules, not exhaustion-related chronic inflammation, as observed in CD8^+^ T cell-induced cardiomyocyte apoptosis in animal models [19].

PD-1 expression reflects T cell function, with low levels indicating early activation and high levels indicating exhaustion [20,21]. In this study, no significant enrichment of PD-1+ T cells was observed, which may imply that the abnormal T cells in DCM are characterized by “overactivation but not exhaustion”. This state may exacerbate myocardial injury by causing continuous secretion of proinflammatory factors (such as IL-17 and IFN-γ) [22]. In addition, PD-1 signaling has a dual role in heart disease, with its inhibitory function alleviating excessive immune responses by T cells [23], while its pathway dysregulation may disrupt the immune tolerance balance [24]. Future research should monitor PD-1 expression over time and analyze the colocalization of PD-L1 with ligands such as PD-L1 in myocardial tissues to understand the PD-1/PD-L1 axis in DCM regulation.

Peripheral blood analysis in DCM patients revealed notable T cell immune dysregulation, characterized by increased CD4^+^ TEM cells and early T cell overactivation (CD69^+^). This immune imbalance extends to the heart, where its detrimental effects are evident. Recent single-cell RNA sequencing [25] has confirmed a positive correlation between CD4^+^ TEM levels in peripheral blood and CD4^+^ TEM infiltration in the myocardial tissue of DCM patients. Both CD4^+^ and CD8^+^ T cell numbers are elevated in the DCM hearts, showing enhanced chemotactic and proinflammatory features. The majority of cardiac T cells express CD69. CD4^+^ TCM cells are found mainly in peripheral blood, whereas CD4^+^ TEM cells are enriched specifically in myocardial tissue, reflecting their distinct roles in immune surveillance and localized immune responses. TCM cells are strategically positioned to oversee the peripheral immune system, whereas TEM cells play a more direct role in targeted immune responses at sites of inflammation or injury.

### 3.2. The Role of Key Diagnostic Markers and Immune Pathways in the Pathogenesis and Progression of DCM

Four diagnostic markers (*LRRTM4*, *PTPN22*, *FAM175B*, and *PROM2*) for DCM were identified with high AUC values, indicating their potential regulatory significance in DCM pathogenesis. These genes contribute to a network involving “immune dysregulation–myocardial injury–fibrotic remodeling” that collectively influences the progression of DCM. Increased *PTPN22* in CD4^+^ T cells from chronic cardiac failure (CHF) patients is associated with increased left ventricular size, a decreased ejection fraction, and exacerbated CHF progression. This is linked to increased early TCR signaling and suppressed Treg differentiation via decreased ZAP-70 tyrosine 292 phosphorylation [26]. This study revealed that high *PTPN22* expression was significantly positively correlated with the abundance of CD4^+^ TCM cells. In DCM, activated TCM cells can stimulate cardiac fibroblast proliferation and collagen deposition via proinflammatory factors such as IL-17 and IFN-γ, contributing to ventricular remodeling [27]. *PTPN22* may influence T cell differentiation, potentially inhibiting Treg cell development and favoring proinflammatory CD4^+^ TCM cells [28]. This study also revealed that high *PTPN22* expression was significantly positively correlated with the abundance of effector memory CD4^+^ TEM cells. In DCM mice, TEM cells from the spleen are pathogenic, inducing myocardial inflammation, fibrosis, and cardiac dysfunction [15]. *PTPN22* abnormalities may enhance TEM cell sensitivity to antigens, increasing activation during chronic inflammation [29].

LRRTM4 is a synaptic adhesion molecule with an unknown cardiac-specific function, yet it has been found to promote cardiac fibroblast activation through Wnt/β-catenin pathway regulation [30]. FAM175B, a key component of the BRCA1-A complex involved in DNA double-strand break repair, was notably upregulated in DCM and potentially linked to the cardiomyocyte response to chronic oxidative stress. The upregulation of *FAM175B* in response to sustained oxidative damage may enhance homologous recombination repair to maintain genomic stability [31]. We found that high expression of *FAM175B* was negatively correlated with the abundance of infiltrating CD56dim NK cells, suggesting that *FAM175B* may aggravate myocardial injury by inhibiting NK cell activity. Studies have shown that NK cell dysfunction is closely related to the progression of viral myocarditis to DCM [32]. In CVB3-induced myocarditis, reduced NK cell function delays viral elimination, causing prolonged myocardial injury and fibrosis [33]. *FAM175B* prevents ATF4 degradation by binding to it, increasing the stability of ATF4 and promoting the expression of CHOP, a key mediator of endoplasmic reticulum stress-induced cell death [34]. If *FAM175B* plays a similar role in NK cells via the ATF4–CHOP pathway, it may affect NK cell survival, activity, or function. The transmembrane glycoprotein PROM2 is notably increased in DCM, potentially influencing stem cell behavior and cardiomyocyte aging. PROM2 competitively binds to the Notch receptor ligand DLL1, blocking the release of the Notch intracellular domain (NICD), resulting in the downregulation of downstream target genes (Hes1 and Hey1) and inhibiting the differentiation of cardiac progenitor cells into functional cardiomyocytes [35]. Elevated *PROM2* levels are observed in aged mouse hearts and in the atria of patients with heart failure with preserved ejection fraction (HFpEF). *PROM2* overexpression induces cardiomyocyte aging, hypertrophy, and stress resistance, while its suppression mitigates these effects [36].

Gene interaction network and pathway analyses revealed that the onset of DCM involves immune homeostasis disruption due to increased *PTPN22* expression. Elevated *PTPN22* levels impede T cell differentiation into Treg cells, increasing proinflammatory TCM cell levels. Consequently, increased secretion of proinflammatory cytokines such as IL-17 and IFN-γ occurs [28]. These cytokines increase *LRRTM4* expression via TLR4/NF-κB signaling [37]. *LRRTM4* not only promotes the transformation of cardiac fibroblasts into myofibroblasts through the Wnt/β-catenin pathway [30] but also exacerbates the generation of reactive oxygen species (ROS) through the mitochondrial pathway, causing double-strand breaks in cardiomyocyte DNA [38]. In response to persistent DNA damage, cardiomyocytes initiate a compensatory repair mechanism, manifested as the upregulation of *FAM175B* expression. As a core component of the BRCA1-A complex, *FAM175B* attempts to maintain genomic stability by enhancing homologous recombination repair [31]. However, when oxidative damage exceeds repair capacity, the ATM/CHK2-p53 pathway is activated, triggering cardiomyocyte senescence and the release of senescence-associated secretory phenotype factors (such as IL-6 and TGF-β1) [39,40]. These factors promote elevated *PROM2* expression in cardiac fibroblasts, hindering cardiac progenitor cell differentiation by inhibiting the Notch pathway [35]. The resulting cycle of “inflammation–damage–fibrosis” increases ventricular wall tension, weakens myocardial contractility, and accelerates cardiomyocyte apoptosis, leading to ventricular dilation and a reduced ejection fraction, which are typical phenotypes of DCM.

### 3.3. Identification of Interaction Patterns Between Diagnostic Genes and Small-Molecule Drugs

This study analyzed the DSigDB database to identify distinct interaction patterns between four diagnostic genes (*PTPN22*, *FAM175B*, *PROM2*, and *LRRTM4*) and small-molecule drugs, offering insights into disease regulatory mechanisms and targeted treatment strategies. Notably, *PTPN22* exhibited significant binding affinity with glibenclamide and nocodazole. Glibenclamide, an ATP-sensitive potassium channel inhibitor, can impede glycolytic metabolic reprogramming by blocking KATP channels in T cells, leading to decreased secretion of proinflammatory cytokines such as IL-1β and TNF-α [41]. Glibenclamide reverses ROS production and caspase-1 activity induced by a high-fat diet, downregulates the TGF-β1-pSmad2/3-NLRP3 signaling pathway, inhibits profibrotic factors such as MMP-9, and alleviates myocardial fibrosis [42]. Nocodazole disrupts immune synapse formation between T cells and antigen-presenting cells (APCs) by inhibiting microtubule polymerization [43] and blocking TCR signaling [44]. This may lead to the blockade of T cell activation and reduce the expansion of proinflammatory Th cells (such as those that secrete IFN-γ and IL-17) [45]. Drugs that interact with *FAM175B* include doxorubicin and mitomycin. Doxorubicin is an important chemotherapeutic drug that causes drug-induced DCM. It regulates genes related to energy metabolism (such as the NAD pathway) [46] and systolic function (such as PGC-1α) [47] in cardiomyocytes, leading to decreased myocardial contractility and ventricular dilation. High *FAM175B* expression in DCM patients may increase doxorubicin sensitivity, necessitating careful evaluation of chemotherapy. Inhibiting *FAM175B* expression could mitigate doxorubicin-induced cardiac damage, but this finding remains to be validated in animal models or clinical trials.

### 3.4. Limitations

Despite providing novel insights into the immune regulatory mechanisms of DCM, this study has key limitations: (1) The initial screening relied on the GSE101585 dataset with a limited sample size; although we validated transcriptional changes in *LRRTM4*, *PTPN22*, *FAM175B*, and *PROM2* via qPCR, independent multicenter cohort validation is still needed to confirm generalizability across diverse populations. (2) This study only validated that CD4^+^ TEM cells are elevated in DCM patient blood samples; whether their infiltration into cardiac tissues is increased has not been validated. (3) Functional predictions were restricted to bioinformatic annotations without mechanistic validation through gene knockdown/overexpression experiments. Future improvements should include validation with larger, multicenter cohorts, confirmation of CD4^+^ TEM cell infiltration in cardiac tissues, and mechanistic validation through gene knockdown/overexpression experiments.

## 4. Materials and Methods

### 4.1. Data Sources and Preprocessing

Supplemental Figure S3 illustrates the entire analytical process in this study. The DCM peripheral blood Bulk RNA-seq dataset GSE101585 [48] from the sequencing platform GPL20301 Illumina HiSeq 4000 (Illumina, Inc., San Diego, CA, USA) *(Homo sapiens)* was obtained from NCBI GEO (https://www.ncbi.nlm.nih.gov/ (accessed on 20 May 2024)) [49]. It consists of 16 samples (8 DCM patients and 8 healthy individuals). The mRNA probe expression matrix file and the platform’s annotation file were downloaded. Probes were converted to gene symbols, and unmatched probes were removed. Average values were calculated for multiple probes targeting the same gene to create a gene expression matrix for analysis. Additionally, the scRNA-seq data GSE145154 [25] for DCM patients and healthy controls, including blood tissue CD45^+^ cell samples from 2 DCM patients (GSM4307524, GSM4307529) and 1 healthy control (GSM4307519), were downloaded from NCBI GEO.

### 4.2. Estimation at the Immune Cell Level

#### 4.2.1. Multi-Algorithm Deconvolution of Immune Cell Subsets

Data samples from GSE101585 were analyzed via the ssGSEA, EPIC, xCell, and CIBERSORT algorithms from the IOBR package (version 2.0.1) [50] to determine the immune cell content. The Wilcoxon test was used to compare the distribution of immune cells between the DCM and healthy groups.

#### 4.2.2. Single-Cell RNA Sequencing Analysis of Immune Cell Subsets

Data samples from GSE145154 were analyzed via the “Seurat” package (version 5.3.0) [51] in R software (version 4.4.2). First, the 10× sequencing data were imported, and a Seurat object was created. Quality control was then performed, and cells with mitochondrial gene expression levels greater than 25% were excluded. The data were subsequently normalized, and 2000 highly variable genes were identified for principal component analysis (PCA) [52]. The number of principal components was determined via an elbow plot, and batch effects were corrected via Harmony (version 0.1.0) [53]. Dimensionality reduction and cell clustering were performed via the t-distributed stochastic neighbor embedding (t-SNE) algorithm [54]. The cells were annotated on the basis of known lineage-specific marker genes and the online database CellMarker (version 2.0) [55].

To investigate the developmental trajectory of CD4^+^ TEM cells in DCM, pseudotime analysis was performed via Monocle 2 (version 2.22.0) [56]. CD4^+^ TEM cells were extracted from Seurat (version 5.3.0) [51] objects, and the raw count matrix was converted to a sparse matrix format. Corresponding cell annotations and gene names were used to construct a CellDataSet object. Size factors and dispersions were estimated using the estimateSizeFactors and estimateDispersions functions. Genes expressed in at least 10 cells were retained for downstream analysis. Differentially expressed genes were identified based on Seurat cluster labels using the differentialGeneTest function, and the top 1000 genes with the lowest q-values were selected as ordering genes. Dimensionality reduction was performed via the DDRTree algorithm, and cell trajectories were inferred via the orderCells function.

### 4.3. Validation at the Immune Cell Level

#### 4.3.1. Research Subjects

To validate the estimation results of immune cell levels via the deconvolution algorithm, a single-center observational study was conducted at West China Hospital of Sichuan University from June 2024 to January 2025 to examine immune cells in patients with DCM and healthy controls. The inclusion criteria for the DCM group were adult patients (≥18 years old) diagnosed with DCM, which is defined by the presence of left ventricular (LV) dilatation and systolic dysfunction, unexplained solely by abnormal loading conditions or coronary artery disease (CAD) [7]. LV dilatation is defined by LV end-diastolic dimensions (LVEDD) or volumes (LVEDV) > 2 z scores above population mean values, corrected for body size, sex, and/or age. For adults, this corresponds to an LVEDD > 58 mm in males and >52 mm in females, and an LVEDV index of ≥75 mL/m^2^ in males and ≥62 mL/m^2^ in females, as measured by echocardiography. LV global systolic dysfunction is defined as an LV ejection fraction (LVEF) < 50%. The exclusion criteria included malignant tumors, autoimmune diseases, allergic diseases, hematological diseases, recent infections, comorbidities, pacemaker implantation, severe liver or kidney dysfunction, recent immunosuppressant or hormone therapy use, pregnancy, lactation, and lack of consent. Healthy controls were adults (≥18 years old) with no history of diseases, medications, infections, or fevers within 1 month. The research protocol was approved by the Biomedical Ethics Committee of West China Hospital of Sichuan University (20200562) on 22 June 2020, and all the subjects provided written informed consent to participate in the study. All procedures performed in the study involving human participants were in accordance with the 1964 Helsinki Declaration and its later amendments.

#### 4.3.2. Multicolor Flow Cytometry for the T Cell Subset

Five milliliters of peripheral venous blood was extracted from both the case and control groups, and coagulation was prevented by inverting the tubes. Ficoll density gradient centrifugation was performed on the human peripheral blood samples to isolate peripheral blood mononuclear cells (PBMCs), which were subsequently washed with PBS containing 2% FBS and resuspended at 1 × 10^6^ cells/mL. The optimal antibody concentrations were determined through preliminary tests, and a negative control was established to eliminate autofluorescence interference. Channel voltages were adjusted via negative and single-staining controls, and a compensation matrix was created for correcting fluorescence spillover. Flow cytometry analysis was conducted using specific fluorescence antibodies (Table 2). The cell subsets were defined (Table 3). The cell suspension was aliquoted into flow tubes and treated with an Fc receptor blocker (Human BD Fc Block, 5 μL/tube) for 10 min at 4 °C in the dark. Surface antibodies were added, and the samples were incubated for 20 min at 4 °C in the dark. The negative control tubes received only PBS. The cells were washed with PBS, fixed, and analyzed within 1 h using a Cytek Aurora CS full-spectrum flow cytometry sorter.

#### 4.3.3. Flow Cytometry Data Analysis and Statistical Processing

Flow cytometry data were analyzed via Flow Jo (version 10.9) software. Statistical analysis was performed via IBM SPSS Statistics (version 26). The Shapiro–Wilk test was used to assess the normality of the data. Normally distributed variables are expressed as the means ± standard deviations, whereas nonnormally distributed variables are expressed as medians (interquartile ranges). Differences between groups were analyzed via a t test or a rank-sum test. Statistical significance was set at *p* ≤ 0.05.

### 4.4. Differentially Expressed Gene Screening

The supplementary file GSE101585_mRNA_Expression_Profiling.xlsx of GSE101585 was downloaded from the GEO database, and this dataset was used as the analysis set. The samples in the analysis set were divided into two groups: DCM and control. Differential expression analysis was performed via DESeq2 (version 1.47.4) [57] in R, where raw count data underwent variance-stabilizing transformation to calculate gene-specific *p*-values and log_2_-fold changes (log_2_FC). DEGs were identified via significance thresholds of *p*-values < 0.05 and |log_2_FC| > 2. These DEGs were subsequently annotated for disease associations through integrated literature mining and DisGeNET (version 24.3) [58] curation. For visualization, log_2_FC values were standardized to z scores via the formula z = (log_2_FC − μ)/σ (where μ represents the mean log_2_FC and σ the standard deviation across all genes), and genes were ranked by log_2_FC values. The resulting plot was generated with ggplot2 (version 3.5.1), displaying this log_2_FC rank order on the X-axis and log_2_FC z scores on the Y-axis. The FPKM data of the analysis set were log_2_-transformed (FPKM + 1) for subsequent analysis.

### 4.5. Construction of the Gene CoExpression Network

The R package WGCNA (version 1.71) [59] was utilized for gene coexpression network analysis on a differential gene expression matrix to detect gene set modules with highly coordinated changes. By employing ssGSEA scores of CD4^+^ T cells as phenotypic traits and MADtop500 data (version 2.1) as the expression matrix, immune cell marker gene modules were identified. The process involved sample clustering, soft threshold power selection for network scale-freeness, hierarchical clustering via the dynamic cutting method for gene dendrograms, and correlation analysis between modules and phenotypes. Modules positively correlated with CD4^+^ T cell ssGSEA scores (*p* < 0.05) were considered key modules related to immune cells and included genes designated immune cell markers.

Intersecting genes were identified by overlapping immune cell marker genes with DEGs. The functional connections among the DEGs were annotated via the STRING database (version 12.0) [60]. A PPI network was constructed and visualized via Cytoscape (version 3.8.2) [61]. Four key indicators (MCC, MNC, degree, and EPC) [62] were calculated for the module genes. Hub genes were determined by selecting the top 50 genes from each algorithm and identifying their intersection.

### 4.6. Machine Learning Feature Selection

The LASSO-logistic [63], SVM-RFE [64], and Boruta [65] algorithms were specifically applied to select the hub genes for further analysis. The characteristic genes were obtained from the intersection of genes identified by these algorithms. LASSO-logistic regression, a method for feature selection and sparse model construction, was implemented via the R package glmnet (version 4.1-8) [66]. SVM-RFE, a feature selection method based on support vector machines, was applied via the R package e1071 (version 1.7-14). The Boruta statistic compares the importance of the original features and the randomly shuffled shadow features in the data and selects the features whose importance is greater than or close to that of the optimal shadow feature, namely, the confirmed/tentative genes, as the marker genes [67]. Boruta analysis was conducted via the R package Boruta (version 8.0.0) [68].

### 4.7. Diagnostic Gene Validation and Immune Association

#### 4.7.1. qPCR for mRNA

To validate the key genes selected through machine learning methods, total RNA was extracted from human peripheral blood CD4^+^ TEM cells of 6 randomly selected DCM patients and 6 healthy controls from the independent clinical cohort (40 DCM patients vs. 40 healthy controls), via TRIzol (Invitrogen, Carlsbad, CA, USA). The relative expression levels of mRNAs were measured via an RT kit (Toyobo, Osaka, Japan) with a SYBR Green Supermix kit (Bio-Rad, Hercules, CA, USA). After a 40-cycle reaction on a Bio-Rad CFX96 RealTime system, the gene expression levels were calculated and normalized to the GAPDH. The relative fold changes were calculated via the 2^−ΔΔCt^ method. The qPCR cycling conditions were as follows: 95 °C for 30 s (initial denaturation), 95 °C for 5 s (denaturation), and 60 °C for 30 s (annealing and extension), with a total of 40 cycles. The experiment was repeated three times to ensure the reliability of the data. The primer sequences are provided in Supplemental Table S1.

#### 4.7.2. Diagnostic Performance and Immune Correlation

Key genes selected via machine learning methods were validated, and their differential expression in the analysis set was assessed via the pROC package (version 1.18.0) [69] in R; ROC curves and gene expression box plots of the hub genes were plotted for diagnostic accuracy assessment. Genes with a *t* test *p* < 0.05 and an AUC > 0.7 were retained for subsequent analysis. A Pearson correlation analysis was conducted to determine the correlations between feature genes and immune cells, which were visualized through scatter plots.

### 4.8. Drug–Gene Interactions

Drug interaction data from DSigDB (version 1.0) [70] were analyzed for diagnostic genes using a significance threshold of FDR < 0.05. The drug–gene regulatory pairs involved targeted activation, inhibitory regulation, and metabolic pathway intervention. An interaction network was constructed in Cytoscape (version 3.8.2) [61], featuring circular nodes for genes and diamond nodes for drugs, with edge weights representing regulatory confidence.

## 5. Conclusions

This study revealed significant enrichment of CD4^+^ T cell subsets in DCM, suggesting their critical role in immunopathology. Four core diagnostic biomarkers (*LRRTM4*, *PTPN22*, *FAM175B*, and *PROM2*) with AUCs > 0.7 were identified through WGCNA and machine learning. Immunological analyses indicated that PTPN22 and FAM175B may modulate TCM/TEM and NK cell functions, respectively. Small-molecule drug prediction highlighted the broad targeting potential of PTPN22, providing key insights for precision diagnostics and therapeutics in DCM.

## Figures and Tables

**Table 1:** Flow cytometric analysis of T cell subsets and activation/exhaustion markers in patients with DCM versus healthy controls.

Parameter	DCM (*n* = 40)	Healthy (*n* = 40)	*p* Value
CD3^+^ T cells (% of lymphocytes)	37.10 ± 16.93	30.16 ± 19.5	0.203
PD-1^+^ exhausted CD4^+^ T cells (% of CD4^+^)	15.95 (8.13, 33.55)	13.8 (8.31, 56.7)	0.773
Th2 cells (% of CD4^+^ T cells)	11.95 (8.32, 18.42)	12 (5.09, 14.77)	0.282
Th1 cells (% of CD4^+^ T cells)	34.4 (13.8, 62.6)	46.4 (40.05, 75.32)	0.034 *
CD4^+^ T cells (% of CD3^+^)	54.15 (41.87, 61.47)	49.55 (4.66, 56.4)	0.043 *
CD4^+^ TCM cells (% of CD4^+^)	21.63 ± 10.93	14.19 ± 7.81	0.013 *
Late-activated CD4^+^ T cells (CD25^+^, % of CD4^+^)	5.24 (0.89, 9.9)	4.44 (2.67, 35.2)	0.268
Naive CD4^+^ T cells (% of CD4^+^)	17.5 (7.28, 28.42)	27.8 (19.97, 36.35)	0.039 *
Early-activated CD4^+^ T cells (CD69^+^, % of CD4^+^)	3.59 (1.39, 19.95)	1.2 (0.62, 1.85)	0.001 *
CD4^+^ TCE cells (% of CD4^+^)	0.77 (0.31, 2.32)	0.81 (0.2, 15.68)	0.485
CD4^+^ TEM cells (% of CD4^+^)	7.54 (4.57, 18.85)	3.96 (1.53, 5.30)	0.001 *
CD8^+^ T cells (% of CD3^+^)	38.74 ± 15.19	24.48 ± 8.34	<0.001 *
Late-activated CD8^+^ T cells (CD25^+^, % of CD8^+^)	5.02 (0.66, 17.98)	2.18 (0.41, 38.93)	0.851
Early-activated CD8^+^ T cells (CD69^+^, % of CD8^+^)	11.65 (5.50, 21.4)	4.61 (3.45, 11.55)	0.012 *
PD-1^+^ exhausted CD8^+^ T cells (% of CD8^+^)	27.25 (5.9, 51.08)	25.85 (16.73, 87.68)	0.223
CD8^+^ TCM cells (% of CD8^+^)	1.07 (0.44, 2.37)	0.95 (0.52, 1.19)	0.912
Naive CD8^+^ T cells (% of CD8^+^)	19.27 ± 11.68	27.98 ± 17.54	0.049 *
CD8^+^ TCE cells (% of CD8^+^)	3.01 (0.44, 42.42)	1.35 (0.43, 3.06)	0.147
CD8^+^ TEM cells (% of CD8^+^)	27.5 (1.11, 31.37)	24.3 (15.92, 31.95)	0.528

Legend: Values are the number of patients (percentage), mean ± standard deviation, or median (interquartile range). *: *p* value < 0.05. Abbreviations: TCM: central memory T cells; TEM: effector memory T cells; TCE: terminal effector T cells.

**Table 2:** Fluorescent-labeled antibodies for flow cytometry and isotype controls.

Cell Surface Markers	Fluorescent Dyes	Clone Number	Species Source	Antibody Vendor
CCR4	BV421	1G1	Mouse	BD Pharmingen
CD45RA	BV510	HI100	Mouse	BD Pharmingen
CD279	BV605	MIH4	Mouse	Biolegend
CD25	BV711	2A3	Mouse	BD Pharmingen
CD3	FITC	7F5	Mouse	Absin
CD45RO	PerCP-Cy5.5	UCHL1	Mouse	BD Pharmingen
CCR7	PE	3D12	Rat	BD Pharmingen
CXCR3	PE-Cy7	1C6/CXCR3	Mouse	BD Pharmingen
CCR6	APC	G034E3	Mouse	BD Pharmingen
CD69	APC-R700	FN50	Mouse	BD Pharmingen
CD8	BUV395	RPA-T8	Mouse	BD Pharmingen
CD4	BUV661	RPA-T4	Mouse	Thermo

**Table 3:** 12-Color flow cytometry panel for T cell subsets and functional markers.

Cell Type/Functional State	Surface Markers
Total T cells	CD3^+^
CD4^+^ T cells	CD4^+^
Tc cells	CD8^+^
Th1 cells	CD4^+^ CXCR3+ CCR6
Th2 cells	CD4^+^ CXCR3− CCR6− CCR4+
Naive T cells	CD45RA+ CD45RO− CCR7+
TCM cells	CD45RA− CD45RO+ CCR7+
TEM cells	CD45RA− CD45RO+ CCR7−
TCE cells	CD45RA+ CD45RO− CCR7−
Early Activation Marker	CD69^+^
Late Activation Marker	CD25^+^
Exhausted T cells	PD-1+ (CD279^+^)

## Data Availability

The original contributions presented in this study are included in the article/Supplementary Material. Further inquiries can be directed to the corresponding authors.

## Supplementary Materials

The following supporting information can be downloaded at: https://www.mdpi.com/article/10.3390/ijms26167806/s1.

## Abbreviations

The following abbreviations are used in this manuscript:

DCM	dilated cardiomyopathy
scRNA-seq	single-cell RNA sequencing
PCA	Principal Component Analysis
t-SNE	t-Distributed Stochastic Neighbor Embedding
TEM cells	effector memory T cells
TCM cells	central memory T cells
TCE cells	terminal effector T cells
WGCNA	weighted gene coexpression network analysis
PPI	protein–protein interaction
LRRTM4	leucine rich repeat transmembrane neuronal 4
PTPN22	protein tyrosine phosphatase non-receptor type 22
FAM175B	family with sequence similarity 175 member B
PROM2	prominin 2
DSigDB	drug signatures database
Th17 cells	type 17 helper T cells
Tc cells	cytotoxic T cells
Th1 cells	type 1 helper T cells
Th2 cells	type 2 helper T cells
FPKM	fragments per kilobase of exon per million fragments mapped
DEGs	differentially expressed genes
NCBI GEO	National Center for Biotechnology Information Gene Expression Omnibus
ssGSEA	single-sample gene set enrichment analysis
EPIC	epigenetic prediction of immune cell composition
xCell	xCell: digitally unlocking the immune microenvironment
CIBERSORT	cell-type identification by estimating relative subsets of RNA transcripts
IOBR	immuno-oncology biological research
PBMCs	peripheral blood mononuclear cells
PBS	phosphate-buffered saline
MCPcounter	microenvironment cell populations counter
SVM-RFE	support vector machine recursive feature elimination
FDR	false discovery rate
cDCs	conventional dendritic cells
HSCs	hematopoietic stem cells
UMAP	uniform manifold approximation and projection
NYHA	New York Heart Association
qPCR	quantitative PCR
HLA-DOB	human leukocyte antigen D obligate beta
CVB3	coxsackievirus B3
ATF4	activating transcription factor 4
NICD	notch intracellular domain
HFpEF	heart failure with preserved ejection fraction
APCs	antigen-presenting cells
PGC-1α	peroxisome proliferator-activated receptor gamma coactivator 1 alpha
LV	left ventricular
CAD	coronary artery disease
LVEDD	left ventricular end-diastolic dimension
LVEDV	left ventricular end-diastolic volume
LVEF	left ventricular ejection fraction
log2FC	log2-fold change
CITE-seq	cellular indexing of transcriptomes and epitopes by sequencing
