# Supplementary material for: CD4+ T Cell Subsets and PTPN22 as Novel Biomarkers of Immune Dysregulation in Dilated Cardiomyopathy
Source: Int J Mol Sci. 2025 Aug 13;26(16):7806. doi: 10.3390/ijms26167806 (PMC12386803; doi:10.3390/ijms26167806)
Supplement: Supplementary file 1 [file ijms-26-07806-s001.zip › figure legends.pdf]

Figure S1. Quality control and principal component analysis (PCA) dimensionality reduction of GSE145154. (A) Frequency histogram of mitochondrial gene percentage (MT%) in single-cell RNA sequencing (scRNA-seq) data. (B) Violin plots of cell cycle-related scores (S.Score and G2M.Score) across different cell clusters in DCM patients and controls. (C) Scatter plots of the correlation between the percentage of mitochondrial genes (percent. MT) and the RNA molecule count (nCount_RNA) and between the gene count (nFeature_RNA) and the RNA molecule count (nCount_RNA). (D) Scatter plots of the correlation between average gene expression and variance of nonvariable and variable genes. (E) PCA plot showing clustering of cells based on different sample sources (DCM1, DCM2, and normal). (F) Violin plots of first principal component (PC1) expression across DCM patients and controls. (G) Expression distribution of PC1 after harmony integration across DCM patients and controls. (H) Heatmap of gene expression for the first four principal components (PC1--PC4) from PCA.

Figure S2 t-SNE visualization of marker gene expression across immune cell subtypes: (A) CCR7, (B) IL7R, (C) NKG7, (D) TRGC2, (E) CD7A, (F) GZMA, (G) LEF1, (H) LYZ, (I) CD27, (J) MZB1, (K) SOX4, (L) CD63.

Figure S3: Flow chart of the entire analysis process.
