# Supplementary material for: CD4+ T Cell Subsets and PTPN22 as Novel Biomarkers of Immune Dysregulation in Dilated Cardiomyopathy
Source: Int J Mol Sci. 2025 Aug 13;26(16):7806. doi: 10.3390/ijms26167806 (PMC12386803; doi:10.3390/ijms26167806)
Supplement: Supplementary file 1 [file ijms-26-07806-s001.zip › Supplemental Table 1.pdf]

Table S1. Sequences of the q-PCR primers.

Genes	Primer sequences (5′-3′)
PTPN22 (human)	F: 5’ ACACACATTTGACATGCCCTC3’<br>R: 5’ CAGAAATTCATTGGCAAACTCCT3’
FAM175B (human)	F: 5’ AGAGCAGGTTCTTCACAAGCA3’<br>R: 5’ AGCACATATTCTAAAGCGTGAGT3’
LRRTM4 (human)	F: 5’ CAGTCGGAGCATTTGTCCTT3’<br>R: 5’ CCTTTTCACCCTGGATGTGCTT3’
PROM2 (human)	F: 5’ CCAAGCCTATCAGCAGTGCAA3’<br>R: 5’ GCTCTGCAACTCCTGCCGTA3’
GAPDH (human)	F: 5’ CTCAAGATCATCAGCAATGCCT3’<br>R: 5’ TGGTCATGAGTCCTTCCACGAT3’
