# Supplementary figures and images for: CD4+ T Cell Subsets and PTPN22 as Novel Biomarkers of Immune Dysregulation in Dilated Cardiomyopathy
Source: Int J Mol Sci. 2025 Aug 13;26(16):7806. doi: 10.3390/ijms26167806 (PMC12386803; doi:10.3390/ijms26167806)

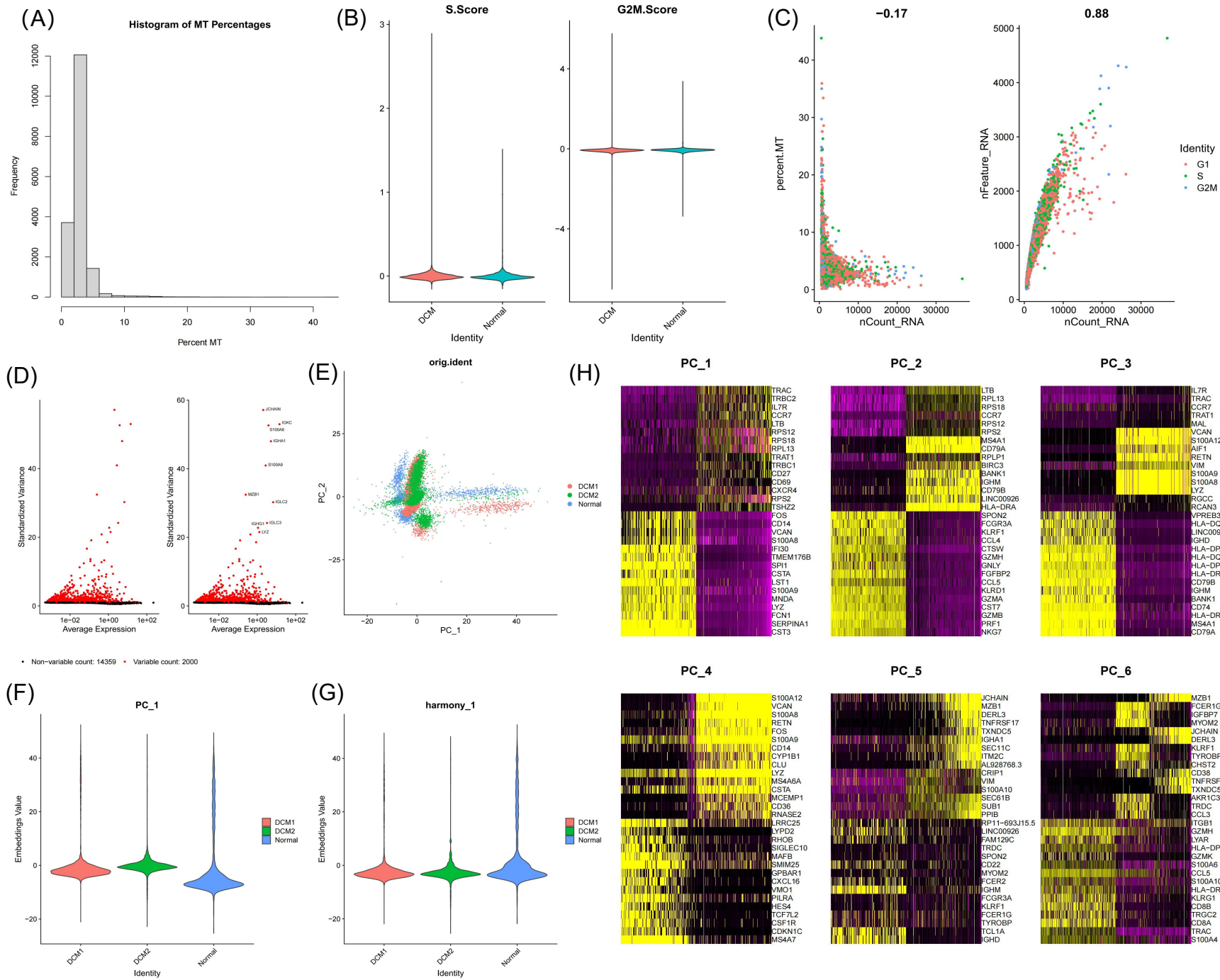

Supplement: Supplementary file 1 [file ijms-26-07806-s001.zip › Figure S1.pdf]

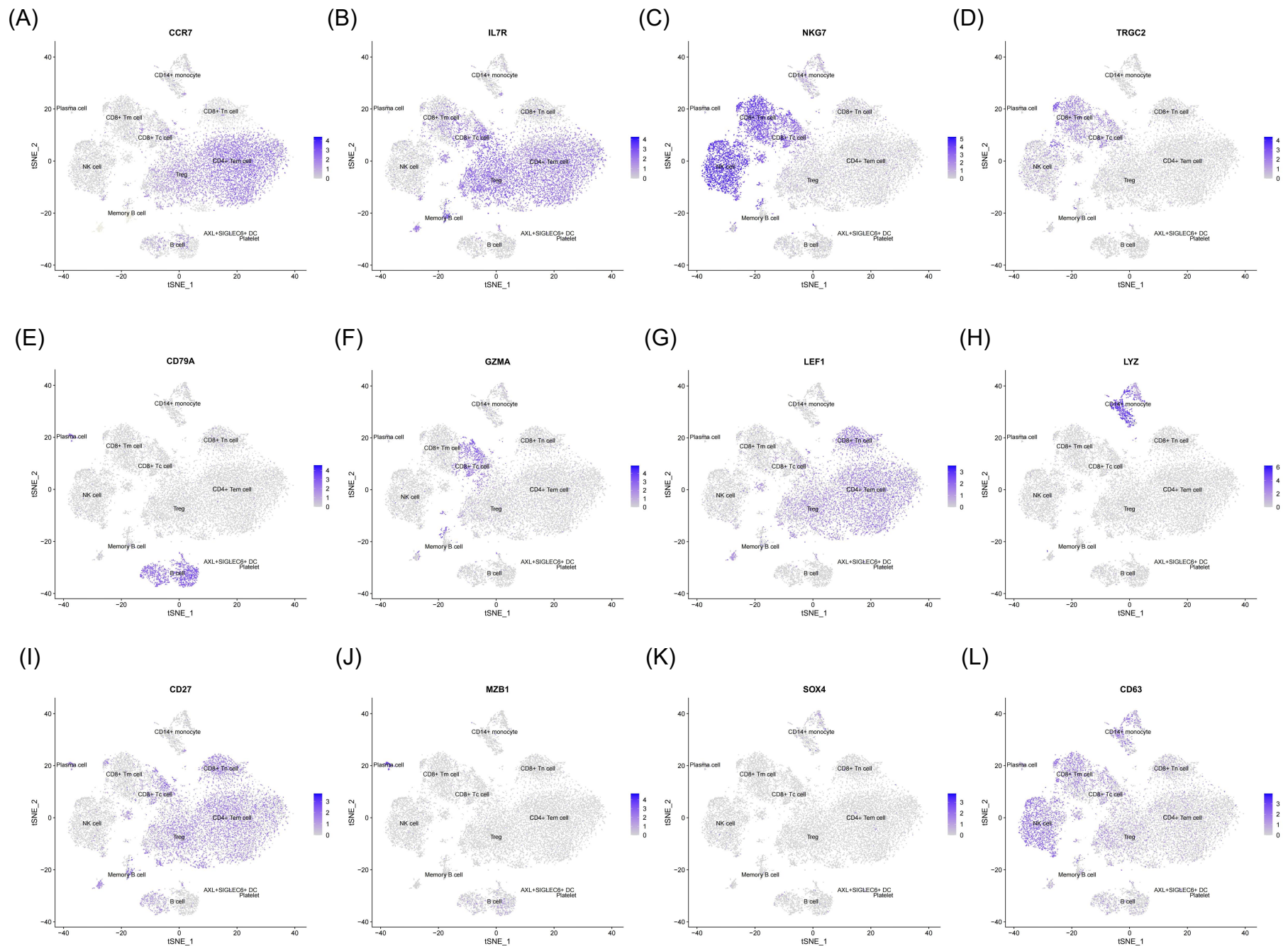

Supplement: Supplementary file 1 [file ijms-26-07806-s001.zip › Figure S2.pdf]

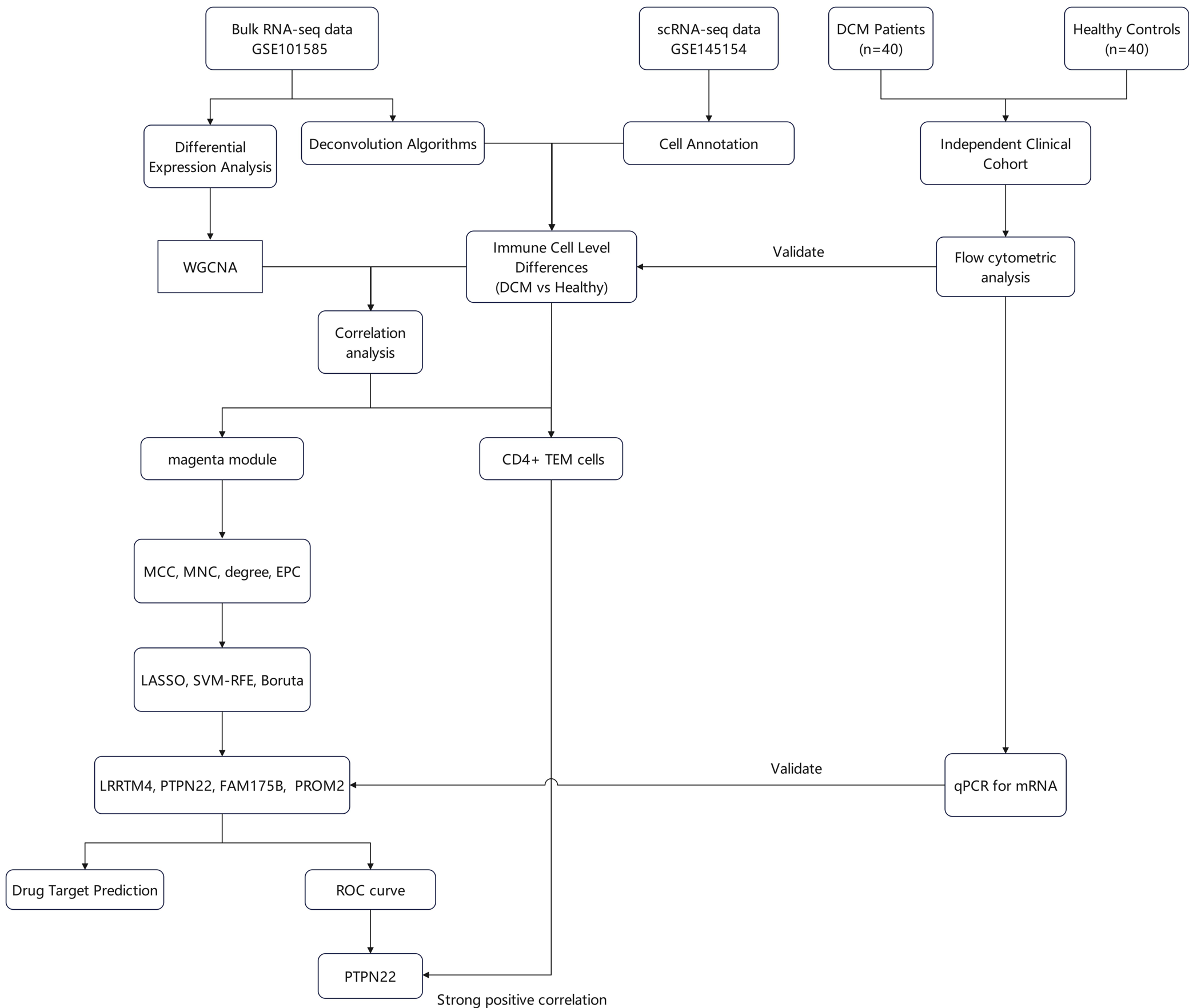

Supplement: Supplementary file 1 [file ijms-26-07806-s001.zip › Figure S3.pdf]
